# Genetic relationships among *Eriobotrya* species revealed by genome‐wide RAD sequence data

**DOI:** 10.1002/ece3.2902

**Published:** 2017-03-21

**Authors:** Xianghui Yang, Samaneh Kazemiani Najafabadi, Muhammad Qasim Shahid, Zhike Zhang, Yi Jing, Weiling Wei, Jingcheng Wu, Yongshun Gao, Shunquan Lin

**Affiliations:** ^1^ State Key Laboratory for Conservation and Utilization of Subtropical Agro‐bioresourcesSouth China Agricultural UniversityGuangzhouChina; ^2^ BGI‐ShenzhenGuangdongChina; ^3^ Environment and Life ScienceCollege of Putian UniversityFuzhouChina

**Keywords:** cluster analysis, *Eriobotrya* genus, phylogenetic relationship, RAD‐seq, SNP

## Abstract

Restriction site‐associated DNA sequencing (RAD‐seq) was used to illuminate the genetic relationships among *Eriobotrya* species. The raw data were filtered, and 221 million clean reads were used for further analysis. A total of 1,983,332 SNPs were obtained from 23 *Eriobotrya* species and two relative genera. We obtained similar results by neighbor‐joining and maximum likelihood phylogenetic trees. All *Eriobotrya* plants grouped together into a big clade, and two out‐groups clustered together into a single or separate clade. Chinese and Vietnam accessions were distributed throughout the dendrogram. There was nonsignificant correlation between genotype and geographical distance. However, clustering results were correlated with leaf size to some extent. The *Eriobotrya* species could be divided into following three groups based on leaf size and phylogenetic analysis: group A and group B comprised of small leaves with <10 cm length except *E. stipularis* (16.76 cm), and group C can be further divided into two subgroups, which contained medium‐size leaves with a leaf length ranged from 10 to 20 cm and a leaf length bigger than 20 cm.

## 1 Introduction

High‐throughput sequencing technologies have revolutionized the genome research in recent years. The field of population genomics is rapidly expanding, and studies are now possible on unprecedented scales even in nonmodel organisms. Restriction site‐associated DNA (RAD‐tag) sequencing can simultaneously detect and genotype thousands of genome‐wide SNPs (Baird et al., 2008; Willing et al., 2011). It is one of the reduced representation methods that sampled a shared set of sites across the genome in many individuals or populations, making population‐scale sequencing possible at a fraction of the cost of whole genome sequencing (Davey et al., 2011). RAD‐Seq is suitable for fine‐scale linkage mapping (Scaglione et al., 2015; Wang, Fang, Xin, Wang, & Li, 2012), population genetics (Hohenlohe et al., 2011; Andersen et al. 2012), phylogenetics, and phylogeography (Cruaud et al., 2014; Rubin, Ree, & Moreau, 2012; Takahashi & Moreno, 2015; Valdisser et al., 2016). RAD‐Seq has also been used to generate large SNP datasets for many plants (Torres‐Martínez & Emery, 2016; Wang, Jin, Zhang, Shen, & Lin, 2015).

The genus *Eriobotrya* Lindl. Rosaceae, subfamily Maloideae, originated in China (Lin & Hu, 2000), and also found in Southeast Asian countries, such as Vietnam, Laos, and Burma. Previous studies showed that there are more than 30 species (varieties or forms) belong to *Eriobotrya* genus. Our research group have collected and conserved 25 species in loquat germplasm resources at South China Agricultural University. Yang, Liu, and Lin (2009) collected *Eriobotrya* germplasm from China and evaluated 18 accessions including 14 species and four varieties by using AFLP markers. ITS (Yang, Lin, & Hu, 2011) and *ADH* gene (Yang, Li, & Zhang, 2012) were used to analyze the phylogenetic relationships in *Eriobotrya* genus. These studies have got some common results, for example, *Eriobotrya* plants were grouped together and separated from other groups; similar species (varieties or forms) always clustered together; *E. japonica*,* E. prinoides,* and *E. malipoensis* were grouped together into the same clade, which showed a close relationship among these species. In addition, *E. seguinii* and *E. henryi* are very different species from other *Eriobotrya* species; they always clustered into the same clade. These previous studies revealed that species with the same morphological characteristics grouped together, while later these species clustered with other species. However, due to the lack of experimental materials and the limited polymorphic sites generated by molecular marker, ITS and *ADH* sequence, the available information is insufficient to evaluate the overall relationships in *Eriobotrya* genus. Especially, the relationships between the native species of Southeast Asian countries and the species originated in China are not yet clear. In this study, RAD‐seq is used to illuminate the genetic relationships between *Eriobotrya* genus, which will provide information on the origin and evolutionary history of different species in this genus, and enable us for the efficient utilization of germplasm resources and better future breeding strategies.

## 2 Materials and methods

### 2.1 Plant materials and DNA isolation

Twenty‐five accessions from 23 *Eriobotrya* species and two relative genera were collected from China, Vietnam, Burma, and Laos and used in this study (Table 1). All the samples were planted at the Loquat Germplasm Center, College of Horticulture, South China Agricultural University, P. R. China. DNA was extracted from young leaves by using a modified cetyltrime thylammonium bromide (CTAB) method as described by Doyle and Doyle (1990) with minor modifications (Liu et al., 2005). After quality assessment, DNA concentrations were adjusted to 100 ng/μl for RAD‐seq library preparation.

**Table 1:** Scientific name, origin, and leaf length of the *Eriobotrya* accessions evaluated in the study

Code	Scientific name	Origin location	The average leaf length(cm)^a^
A1	*Raphiolepis indica* Lindl.	Huadu, Guangdong, China	5.28 ± 0.52
A2	*Photinia serrulata* Lindl.	Kunming, Yunnan, China	6.64 ± 0.68
A3	*E. angustissima* Hook.	Dalat, Vietnam,	9.85 ± 0.24bc
A4	*E. stipularis* Craib	Dalat, Vietnam,	16.76 ± 0.47bc
A5	*E. seguinii* Card.	Baishe Guangxi, China	4.45 ± 0.93c
A6	*E. henryi* Nakai	Chengjiang Yunnan, China	9.27 ± 0.56bc
A7	*E. kwangsiensis* Chun.	Xiangzhou Guangxi, China	13.93 ± 1.23bc
A8	*E. prinoides* var*. laotica* Vidal	Thong Hai Hin, Laos,	11.06 ± 1.13bc
A9	*E. bengalensis* f*. angustifolia* Vidal	Kunming Yunnan, China	11.73 ± 0.16bc
A10	*E. deflexa* f*. koshunensis* Nakai	Taiwan, China	15.17 ± 2.64bc
A11	*E. fragrans* Champ.	Ruyuan Guangdong, China	16.24 ± 2.14bc
A12	*E. prinoides* Rehd. and Wils.	Shiping Yunnan, China	12.99 ± 1.03bc
A13	*E. deflexa* f*. buisanensis* Nakai	Taiwan, China	13.39 ± 0.21bc
A14	*E. cavaleriei* Rehd.	Lianzhou Guangdong, China	16.84 ± 1.69bc
A15	*Unknow speices*	Jianfengling Hainan, China	16.56 ± 1.95bc
A16	*E. *× *daduheensis* H.Z.Zhang ex W.B.Liao, et al.	HanyuanSichuan, China	14.96 ± 2.03bc
A17	*E. bengalensis* f. Hook.	Lushui Yunnan, China	13.36 ± 1.23bc
A18	*E. deflexa* Nakai	Taiwan, China	15.93 ± 1.78bc
A19	*E. petiolata* Hook.	Pyi Oo Lwi, Burma,	18.42 ± 1.66bc
A20	*E. salwinensis* Hand‐Mazz	Pianma Yunnan, China	17.21 ± 3.97bc
A21	*E. serrate* Vidal	Jinhong Yunnan, China	22.76 ± 0.30ab
A22	*E. japonica* Lindl.	Yangshan Mountain Guangdong, China	22.41 ± 2.51ab
A23	*E. elliptica* var. *petelotii* Vidal	Lào Cai, Vietnam,	33.49 ± 1.35a
A24	*E. elliptica* Lindl.	Shiping Yunnan, China	34.65 ± 1.10a
A25	*E. malipoensis* Kuan	Malipo Yunnan, China	35.78 ± 0.25a

a The data of leaf length represent 2‐year field investigations. Any two means not sharing a letter in common differ significantly at *p* ≤ .01.

### 2.2 The investigation of leaf length

Thirty mature leaves (from five to 10 individuals) were sampled to measure the leaf length. The leaf investigation was carried out for consecutive years. Significant difference analysis (SPSS) was performed at 0.01 level.

### 2.3 RAD‐seq library preparation

RAD‐seq library was prepared by using 5 units of *Nsi*I and *Mse*I (NEB, USA) to digest 1,000 ng genomic DNA per sample at 37°C for 2 hr in a 50 μl reaction volume and then inactivate enzyme at 80°C for 20 min (Zhang et al., 2012). The ligation reaction was performed with 8 μl of 0.1 μmol/L modified Solexa P1 Adaptor and 1 μl of 10 μmol/L Solexa P2 Adaptor (Illumina, USA), along with 30 μl of the digested DNA sample, 5 μl of 10 mmol/L ATP (Promega, USA), 10× NEB Buffer 3, 1.25 μl concentrated T4 DNA ligase (400 U/μl) (NEB, USA), and 2.75 μl H_2_O at room temperature for overnight. P1 and P2 adaptor sequences were as follows: P1 top: 5′‐GATCTACACTCTTTCCCTACACGACGCTCTTCCGATCTxxxxxTGCA‐3′ (xxxx indicates barcode), P1 bottom: 5′‐yyyyAGATCGGAAGAGCGTCGTGTAGGGAAAGAGTGTAGATC‐3′ (yyyy indicates reverse complement of barcode); P2 top: 5′‐TAGATCGGAAGAGCACACGTCTGAACTCCAGTCACCTTGTAATCAGAACAA‐3′, P2 bottom:5′‐CAAGCAGAAGACGGCATACGAGATTACAAGGTGACTGGAGTTCAGACGTGTGCTCTTCCGATC‐3′. After ligation, each DNA sample was heat inactivated at 65°C for 20 min and then purified using the QIA quick PCR purification kit (Qiagen, Germany). In order to enrich the adapter‐modified fragments, purified product was amplified with 25 μl Phusion Master Mix (NEB, USA), 2 μl of 10 μmol/L modified Solexa amplification primer mix (Illumina, USA), and add H_2_O to 50 μl. Phusion PCR preceded following product guidelines (NEB, USA) for 18 cycles. PCR products were electrophoresed on a 1% agarose gel (Sigma, USA), and DNA fragments between 200 and 500 bp were isolated using a Min Elute Gel Extraction kit (Qiagen, Germany) and diluted to 10 μmol/L for Illumina HiSeq2000 sequencing using single‐end sequence.

### 2.4 Quality filtering and SNP calling

Low‐quality reads (Q score < 20) and reads with contamination were filtered out; reads were trimmed to 84 nucleotides to remove flanking barcode sequences. All reads were pooled and used for a de novo assembly and SNP calling in ustacks (STACKS pipeline, Catchen, Amores, Hohenlohe, Cresko, & Postlethwait, 2011). We set a minimum stack size of 5 reads (‐m) and maximum distance between stacks (‐M) within a locus as 2. Population snps were filtered reserving more than half of the samples have snp information. The Illumina data set has been deposited in NCBI sequence read archive (SRA) under accession number PRJNA342569.

Neighbor‐joining and maximum likelihood phylogenetic trees were constructed by Treebest software, and bootstrap replicates were set to 1,000.

## 3 Results

### 3.1 The investigation of leaf length

The leaf length of *Eriobotrya* plants was ranged from 4.45 cm (*E. seguinii*.) to 35.78 cm (*E. malipoensis*) (Table 1). We found three groups: (1) Three species were found with <10 cm leaf length, including, *E. seguinii*., *E. henryi,* and *E. angustissima*, but only *E. seguinii* showed significant difference from other species; (2) A group of 15 species having leaf lengths between 10 and 20 cm was found, and there was nonsignificant difference among these species; (3) Five species exhibited >20 cm leaf length and grouped together. *E. ellipticavar E. petelotii*,* E. elliptica,* and *E. malipoensis* were found to be significantly different from the rest of species at the 0.01 significance level.

### 3.2 RAD‐tag sequencing and SNPs calling

We got 221 million clean reads by using Illumina HiSeq2000, after removing low‐quality reads (Q score < 20), and ambiguous reads with incorrect barcodes. The sequencing quality scores of 20 (Q20), which represent an error rate of 1 in 100, with a corresponding call accuracy of 99%, of all samples were more than 97.6%, indicating that the sequencing quality was good. Of these high‐quality reads, the highest reads (37.71 million reads) were detected in *E. bengalensis* f*. angustifolia,* and the lowest reads (1.96 million reads) were found in *E. japonica*, with an average read number of 8.84 million per accession.

We obtained a total of 1,983,332 SNPs, among them, 1,720,528 and 262,804 SNPs were homozygous and heterozygous, respectively (Table 2). The average number of detected SNPs was 79,333 per accession. The highest number of SNPs (123,089) was detected in *E. bengalensis*, while the lowest number of SNPs (47,351) was detected in *Raphiolepis indica*.

**Table 2:** The SNPs number and information by RAD‐seq

Code^a^	Clean reads number (M)	Q_20_ (%)	Homo SNPs	Hete SNPs	Total SNPs
A1	7.20	98.37	40,776	6,575	47,351
A2	4.87	98.40	43,511	4,127	47,638
A3	4.94	98.26	58,397	9,344	67,741
A4	12.74	97.65	58,639	6,734	65,373
A5	10.54	98.21	59,671	9,566	69,237
A6	9.06	98.22	57,222	8,189	65,411
A7	2.01	98.38	71,969	5,137	77,106
A8	12.74	97.65	81,180	8,516	89,696
A9	3.24	98.36	85,107	18,937	104,044
A10	37.71	98.26	49,892	4,268	54,160
A11	8.08	98.11	82,754	11,621	94,375
A12	5.41	98.40	77,979	6,368	84,347
A13	10.28	98.57	57,312	7,967	65,279
A14	12.17	98.41	85,147	12,128	97,275
A15	10.20	98.28	78,542	9,318	87,860
A16	9.23	98.45	82,320	24,291	106,611
A17	5.97	98.25	95,229	27,860	123,089
A18	16.79	98.47	56,032	6,548	62,580
A19	5.92	98.20	77,332	21,374	98,706
A20	4.94	98.20	50,168	7,369	57,537
A21	4.56	98.37	78,693	12,468	91,161
A22	1.96	98.35	64,893	6,755	71,738
A23	7.58	98.23	81,989	11,729	93,718
A24	3.32	98.58	78,058	10,091	88,149
A25	2.75	98.42	67,626	5,524	73,150
Average	8.84	98.26	68,821	10,512	79,333
Total	220.97		1,720,528	262,804	1,983,332

a Please see Table 1 for scientific names of accessions.

### 3.3 Phylogenetic relationship revealed by RAD‐seq

Although two different approaches were used to construct the phylogenetic tree, similar results were obtained by both methods. All *Eriobotrya* plants grouped together and formed a bigger clade. In this clade, *E. seguinii* and *E. henryi* were always grouped together before clustering with other species. The same situation was found between *E. stipularis* and *E. angustissima*. The rest of 19 *Eriobotrya* plants grouped together. The major difference between two phylogenetic trees was the clustering of out‐groups. In NJ tree, two out‐groups, *Rhaphiolepis indica* and *Photinia serrulata*, grouped together and formed a clade, while they formed a separate clade in MJ tree. Interestingly, out‐groups were clearly separated from *Eriobotrya* plants.

**Figure 1:**
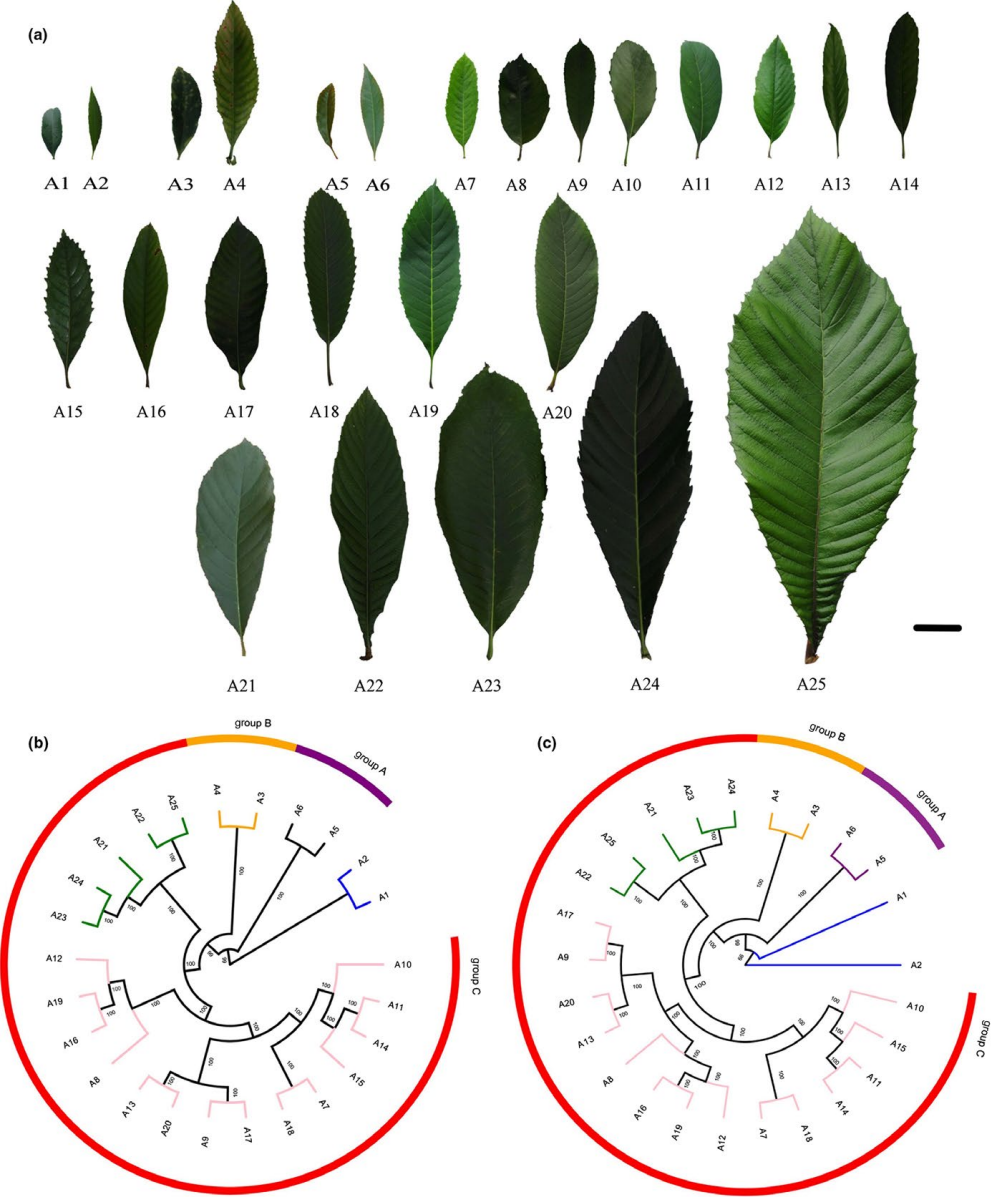
Leaf size and phylogenetic trees of *Eriobotrya* species and two relative genera. (a) The leaf size of 23 *Eriobotrya* species and two relative genera. Bar: 2 cm. (b,c) are neighbor‐joining and maximum likelihood phylogenetic trees of 23 *Eriobotrya* species and two relative genera by RAD‐seq. Node support is given as the maximum parsimony bootstrap value. Group C consists of two subgroups and marked as green and pink, respectively

The phylogenetic analysis divided 23 *Eriobotrya* plants into three major groups, A, B, and C, which were correlated with the size of leaves. Group A consisted of two species from China and both of them have the smallest leaves in *Eriobotrya* genus. Group B included two species from Vietnam and both have small, long, and narrow leaves. Group C was comprised of most of the Chinese species (16 of 19), and other three species were from Vietnam, Laos, and Burma. Group C could be divided into two subgroups, one subgroup included five species, and they have the biggest leaves. In contrast, other 14 species comprised the other subgroup, and they all have medium‐size leaves (Figure 1).

Although we detected variations in the phylogenetic analysis, some results were consistent with the previous studies (Yang, Li, Liu, & Lin, 2009; Yang, Liu, et al., 2009; Yang et al., 2011, 2012). For example, the accessions belonging to the same species were classified into the same cluster, such as *E. bengalensis* and *E. bengalensis* f*. angustifolia*. Notably, *E. japonica* and *E. malipoensis* were always grouped together before clustering with other species. The same situation was found between *E. seguinii* and *E. henryi* and between *E. fragrans* and *E. cavaleriei*. However, *E. defleax*,* E. deflexa* f*. buisanensis* and *E. defleax* var. *koshunensis* belong to the same species, but clustered into different groups.

## 4 Discussion

Next‐generation sequencing technologies have facilitated the study of organisms on a genome‐wide scale. RAD‐seq allows sampling sequence information at reduced complexity across a target genome using the Illumina platform. Paired‐end RAD‐seq provides a large number of informative genetic markers in reference as well as nonreference organisms (Willing et al., 2011). In the present study, we detected 1,983,332 polymorphic SNPs through RAD‐seq technology, which was much higher than AFLP (282 polymorphic locus; Yang, Liu, et al., 2009) and RAPD (232 polymorphic bands; Yang, Li, et al., 2009).

There are more than 30 species in *Eriobotrya* genus, which are largely distributed in China and Southeast Asian countries. In order to analyze the genetic relationships between these species, a phylogeny was constructed using NJ and ML approach. Here, the 23 *Eriobotrya* plants were clustered into three major groups (A, B, and C). The results showed that the Chinese and Vietnam accessions were distributed throughout the dendrogram. We did not find a correlation between genotype and geographical distance However, it is worthwhile to mention that the clustering of accessions was highly correlated with the size of leaves, and the *Eriobotrya* plants were divided into three groups (A, B, and C). Cluster analysis showed that *E. seguinii* and *E. henryi* belong to the same group (group A), which was in agreement with the morphological analysis of these plants. These two species have the smallest leaves, which were quite different from other species. The average leaf length was 9.27 cm for *E. henryi* and 4.45 cm for *E. seguinii*. *E. angustissima* and *E. stipular* were clustered into another group (B), and the average leaf length was 9.85 cm for *E. angustissima* and 16.76 cm for *E. stipularis*. Most of the species (3 of 4) in group A and B have the smaller leaves (<10 cm) than other groups, except *E. stipularis*, which had leaves larger than 10 cm.

Group C was divided into two subgroups. Among these 19 species, *E. japonica*,* E. malipoensis*,* E. serrate*,* E. elliptica,* and *E. elliptica* var*. petelotii* were clustered together. All these species had larger leaves, >20 cm, especially *E. malipoensis*, which had leaves up to 35.78 cm. The rest of the 14 species had medium‐sized leaves (i.e., the average leaf length was ranged from 10 to 20 cm) and they clustered together. Previous studies carried out the classification of *Eriobotrya* plants according to the presence or absence of trichromes on adaxial surface of leaves (Yu, 1974) and flowering time (Zhang, 1996; ). The presence or absence of trichromes on adaxial surface of leaves can be the dichotomous characters or as one of the morphological characters of loquat but cannot be the criteria for the classification of *Eriobotrya* plants. However, the flowering time can be one of the main criteria for the classification of *Eriobotrya* plants, but this was performed with the little knowledge about *Eriobotrya* plants. Recently, the flowering time of some loquat species exhibited abundant variations under different geographical conditions (Lin & Liu, 2016). For example, spring is the flowering time of *E. deflexa* Nakai f. *koshunensis* (originated Taiwan), while the flowering appeared during autumn/winter season when introduced to Guangzhou, China. Therefore, the presence or absence of trichromes on adaxial surface of leaves and flowering time are not suitable criteria for the classification of the *Eriobotrya* plants. By RAD sequencing, the analysis results showed that the *Eriobotrya* plants may be classified according to the leaves size, with the combination of other characters. Here, *Eriobotrya* plants were divided into three categories, small leaves, medium leaves, and large leaves. These results were in accordance with the preliminary classification proposed by Yang and Lin (2007).

It has been universally recognized that common loquat (*E. japonica*) is native to China, and most of the *Eriobotrya* species are distributed in China. However, some Southeast Asian countries are also the distribution centers of *Eriobotrya* species. Our study clearly showed that two varieties native to Southeast Asian countries, *E. elliptica* var. *petelottii* and *E. prioides* var. *laotica*, were classified as Chinese species *E. elliptica* and *E. prinoides*, respectively. The results demonstrated that these two varieties may have close genetic relationship with Chinese species; however, whether these plants are derived from the *Eriobotrya* in China is still uncertain.

## 5 Conclusion

This study revealed the genetic relationships among *Eriobotrya* species by restriction site‐associated DNA sequencing (RAD‐seq). A total of 1,983,332 SNPs were obtained from 23 *Eriobotrya* species and two relative genera. We obtained similar results by neighbor‐joining and maximum likelihood phylogenetic trees. Our results are reliable, all *Eriobotrya* plants grouped together into a big clade, and two out‐groups clustered together into a single or separate clade. Chinese and Vietnam accessions were distributed throughout the dendrogram. The clustering results were correlated with leaf size, and the *Eriobotrya* species could be divided into three groups based on leaf size.

## Conflict of interest

None declared.
